# Acute Paraplegia Result from Spinal Ischemia Nine Years After Hybrid Total Arch Repair with Frozen Elephant Trunk: A Case Report

**DOI:** 10.21470/1678-9741-2022-0327

**Published:** 2023-07-18

**Authors:** Zeqin Xu, Zhu Tong, Jianming Guo, Yixia Qi, Liqiang Li, Lianrui Guo

**Affiliations:** 1 Department of Vascular Surgery, Xuan Wu Hospital and Institute of Vascular Surgery, Capital Medical University, Beijing, People’s Republic of China

**Keywords:** Spinal Cord Ischemia, Paraplegia, Perfusion, Blood Vessel Prosthesis Implantation, Drainage, Ischemia

## Abstract

Spinal cord ischemia due to decreased cord perfusion is a devastating complication in patients with thoracoabdominal dissection following frozen elephant trunk (FET) repair surgery. However, rare occurrence of spinal cord ischemia leading to paraplegia after long-term follow-up of FET repair has been reported. Here, we describe a case of spinal cord ischemia resulting in paraplegia nine years after hybrid total arch repair with FET. Cerebrospinal fluid drainage and serial treatment were utilized to decrease intraspinal pressure and increase blood flow to the spinal cord. Three months after the onset of paraplegia and with treatment and rehabilitation, the patient recovered to walk.

## INTRODUCTION

**Table:** 

Abbreviations, Acronyms & Symbols	
CSFD	= Cerebrospinal fluid drainage
CTA	= Computed tomography angiography
FET	= Frozen elephant trunk
SCI	= Spinal cord ischemia
TAAD	= Type A aortic dissection

Spinal cord ischemia (SCI) is a feared complication following total arch repair for acute type A Stanford aortic dissection. In meta-analysis studies, SCI occurred in 3.5-5.1% of patients who underwent frozen elephant trunk (FET) repair^[1-3]^. As an effective treatment strategy to prevent SCI, cerebrospinal fluid drainage (CSFD) could reduce the incidence of SCI to just 2.3%, and adjunctive treatment to decrease SCI with CSFD includes motor-evoked potential monitoring, hypothermia, distal aortic perfusion, and revascularization of segmental arteries^[4]^. Here, we describe a case of SCI nine years after FET that was reversed using a dedicated spinal cord rescue protocol. The patient approved this study and the publication of his treatment.

## CASE PRESENTATION

**Fig. 1:**
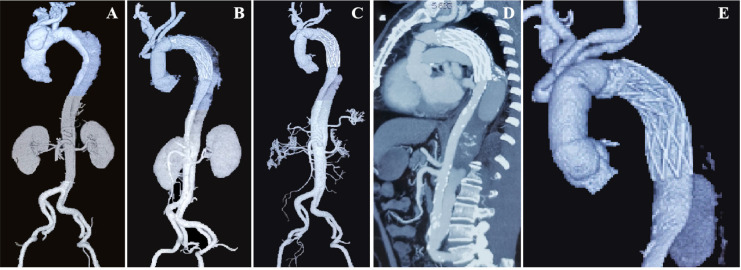
Computed tomography angiography images. (A) Before operation, (B) after operation, (C) one year of follow-up, and (D) and (E) eight years of follow-up.

A 59-year-old man complained of bilateral lower extremity weakness, sensory loss, and dysuria with no fever, no dyspnea, no dizziness, no headache, no speech disorder, and no confusion. Computed tomography angiography (CTA) examination showed aortic dissection after aortic arch replacement (DeBakey III). Past medical history showed that the patient underwent FET because of aortic dissection nine years before (Figure 1A). After the operation, he took oral antiplatelet drugs, including aspirin and clopidogrel, for a long time; aspirin was discontinued one year before because of gastric bleeding. Physical examination showed that the patient was conscious, and a protruded tongue without deviation. His bilateral pupils were sensitive to light reflex with equal size and circle. His sensory level was at T8, both upper limbs muscle strength was grade 5, and bilateral lower limb muscle strength was grade 0. His bilateral femoral arteries, popliteal arteries, dorsal pedis arteries, and posterior tibial arteries could be touched.

**Fig. 3:**
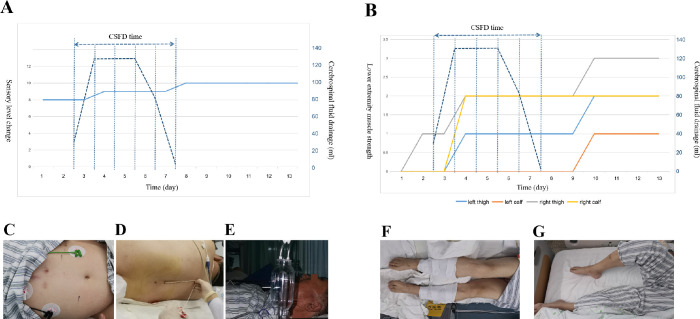
(A) Sensory change before and after cerebrospinal fluid drainage (CSFD). (B) Lower extremity muscle strength change before and after CSFD. (C) Sensory level improved to T10 after CSFD. (D) CSFD process. (E) Cerebrospinal fluid pressure was 10 cmH2O. (F) Patient’s lower extremity muscle showed no activity before CSFD. (G) Patient’s lower extremity muscle recovered partial activity after CSFD.

Our protocol for spinal cord rescue is administration of dexamethasone (10 mg once a day); systolic blood pressure was maintained at 160-170 mmHg, and activated partial thromboplastin time was maintained at 60-70 s after heparin anticoagulation. Cerebrospinal fluid pressure was maintained at 10 cmH2O after CSFD, and the drainage volume was maintained between 100 and 200 ml in 24 hours (Figure 3D, E). After serial treatment, his sensory level recovered to T10 (Figure 3A, C), his muscle strength of left lower limb recovered to grade 2, and right lower limb recovered to grade 3 (Figure 3B, F, G); finally, the patient recovered to walk after three-month rehabilitation.

## DISCUSSION

Both surgical and endovascular repair of an aortic aneurysm or dissection can lead to infarction of the spinal cord because the vascular supply of the spinal cord largely originates directly from the aorta^[5]^. The FET repair has been utilized to treat acute Type A aortic dissection (TAAD). SCI is a devastating complication following FET repair and it can lead to severe disability, including paraplegia. In the literature, there is great variation in SCI rates^[6,7]^. In meta-analysis studies, SCI occurred in 3.5-5.1% of patients who underwent FET^[1-3]^. Postoperative paraplegia or paraparesis have been observed in 1.7-5.5% of patients^[6,8,9]^. A recent report about fenestrated endovascular aneurysm repair and branched endovascular aneurysm repair reported a total incidence of paraplegia in 4% and paraparesis in 13.7% of the patients^[10]^.

**Fig. 2:**
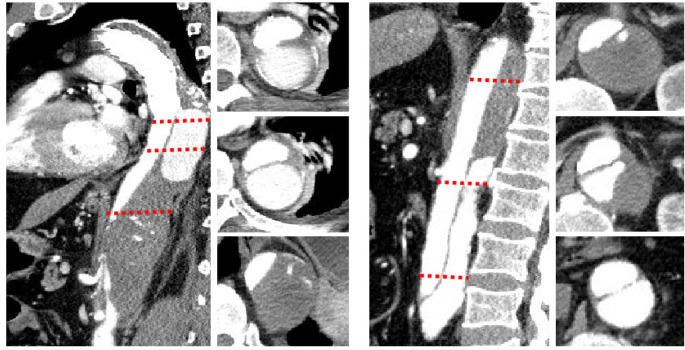
Computed tomography angiography image after onset of paraplegia in our emergency room

It has been reported that paraparesis or paraplegia generally occurs in the early postoperative hours after clinical surveillance. A case report describes delayed-onset paraplegia 12 days after hemiarch replacement for acute TAAD^[11]^. Here, we describe a case of SCI resulting in paraplegia nine years after FET repair. A dedicated SCI protocol was applied to rescue the patient from paraplegia. CTA examination of aortic artery showed the contrast medium filling in the distal anchor position of false lumen of aortic artery (Figure 2). Moreover, this position of the aortic artery slowly increases after the operation (Figures 1B, C, and E). This may result in spinal artery ischemia and paraplegia. In addition to the thrombosed false lumen and stent graft by itself, spinal cord perfusion was reported to depend on the spinal arterial blood pressure. Investigators have reported that CSFD can be positioned on the first postoperative day or at the onset of symptoms.

In this report, our protocol of spinal cord rescue is administration of dexamethasone (10 mg per day), systolic blood pressure maintained at 160-170 mmHg, and placement of spinal drain (cerebrospinal fluid pressure was maintained at 10 cmH₂O and 24-hour drainage volume at 100 to 200 ml). After the serial treatment, sensory level and lower extremity muscle strength of the patient improved, and these may result from the improvement of spinal artery perfusion.

## CONCLUSION

Our SCI rescue protocol was successful in reversing paraplegia in this patient. For paraplegia patients with follow-up imaging demonstrating progressive enlargement of false lumen after FET repair, early CSFD maybe a be beneficial treatment for recovery.
